# Blunted Nocturnal Salivary Melatonin Secretion Profiles in Military-Related Posttraumatic Stress Disorder

**DOI:** 10.3389/fpsyt.2019.00882

**Published:** 2019-12-06

**Authors:** Michel A. Paul, Ryan J. Love, Rakesh Jetly, J. Donald Richardson, Ruth A. Lanius, James C. Miller, Michael MacDonald, Shawn G. Rhind

**Affiliations:** ^1^Defence Research & Development Canada, Toronto Research Centre, Operational Health and Performance Section, Toronto, ON, Canada; ^2^Directorate of Mental Health, Canadian Forces Health Services, Ottawa, ON, Canada; ^3^Department of Psychiatry, Western University, London, ON, Canada; ^4^Department of Psychiatry and Behavioural Neurosciences, McMaster University, Hamilton, ON, Canada; ^5^Operational Stress Injury Clinic, Parkwood Institute, London, ON, Canada; ^6^MacDonald Franklin Operational Stress Injury Research Centre, Lawson Research Institute, London, ON, Canada; ^7^Department of Neuroscience, Western University, London, ON, Canada; ^8^Department of Life Sciences, Texas A&M University Corpus Christi, Corpus Christi, TX, United States

**Keywords:** sleep disturbance, melatonin, post-traumatic stress disorder, dim light melatonin onset, circadian, saliva

## Abstract

**Background:** Sleep disturbances are a hallmark of posttraumatic stress disorder (PTSD), yet few studies have evaluated the role of dysregulated endogenous melatonin secretion in this condition.

**Methods:** This study compared the sleep quality and nocturnal salivary melatonin profiles of Canadian Armed Forces (CAF) personnel diagnosed with PTSD, using the Clinician Administered PTSD Scale (CAPS score ≥50), with two healthy CAF control groups; comprising, a “light control” (LC) group with standardized evening light exposure and “normal control” (NC) group without light restriction. Participants were monitored for 1-week using wrist actigraphy to assess sleep quality, and 24-h salivary melatonin levels were measured (every 2h) by immunoassay on the penultimate day in a dim-light (< 5 lux) laboratory environment.

**Results:** A repeated measures design showed that mean nocturnal melatonin concentrations for LC were higher than both NC (p = .03) and PTSD (p = .003) with no difference between PTSD and NC. Relative to PTSD, NC had significantly higher melatonin levels over a 4-h period (01 to 05 h), whereas the LC group had higher melatonin levels over an 8-h period (23 to 07 h). Actigraphic sleep quality parameters were not different between healthy controls and PTSD patients, likely due to the use of prescription sleep medications in the PTSD group.

**Conclusions:** These results indicate that PTSD is associated with blunted nocturnal melatonin secretion, which is consistent with previous findings showing lower melatonin after exposure to trauma and suggestive of severe chronodisruption. Future studies targeting the melatonergic system for therapeutic intervention may be beneficial for treatment-resistant PTSD.

## Introduction

Degraded mental health remains prevalent and impactful in military personnel (1, 2), and has significant implications for operational readiness and force sustainability (3–5). Posttraumatic stress disorder (PTSD) is a chronic and disabling neuropsychiatric condition that develops in a subset of individuals after exposure to traumatic or extremely stressful events (6). According to the Diagnostic and Statistical Manual 5^th^ Edition (DSM-5) criteria (7), PTSD is characterized by an array of affective, cognitive and attention disturbances, that manifest as four clusters of symptoms. By the very nature of their profession, military personnel can be exposed to traumatic stressors (i.e., combat, injury, witnessing suffering, and/or death) and consequently are at high risk for developing PTSD following war-zone deployment(s) (8–11). Effective prevention and treatment strategies for military-related PTSD are hampered by a lack of understanding of the basic pathophysiologic mechanisms/markers across different stages of disease progression (12–14). Moreover, the management of treatment-resistant PTSD presents a complex clinical challenge (15, 16) and many patients may continue to endure a heavy symptom burden, even despite the best available treatments (17).

Sleep disturbances are considered hallmark symptoms of PTSD and may contribute significantly to the development and maintenance of PTSD (18). Indeed, sleep complaints remain the most common presenting symptoms and subjective complaints among people with PTSD (19). Healthy sleep is defined by predictable, sufficient restorative sleep (20). These needs are disrupted in combat-exposed soldiers and veterans with PTSD (21, 22). According to the DSM-5, sleep disturbance and recurrent trauma-content nightmares are core hyperarousal and re-experiencing symptoms of PTSD (23). About 70% of individuals with PTSD have co-occurring sleep problems, reporting greater trouble initiating and maintaining sleep, early morning awakening, nightmares, nocturnal panic attacks, and other parasomnias (24). Distressed awakenings and inability to return to sleep, with or without recalled trauma nightmares, may be the most common symptom motivating combat veterans to seek treatment for PTSD (22, 25).

Relevant preclinical and clinical literature has neglected the potentially important pathophysiological role of circadian rhythm disruption in PTSD (26). Despite considerable research examining the association between sleep and PTSD (27), few studies have attempted to identify specific underlying biological/circadian mechanisms that may account for this relationship (28, 29). McFarlane et al. (30) found lower urinary melatonin levels after trauma exposure, predicting a higher risk for PTSD, while van Liempt et al. (31) reported undisturbed nocturnal plasma melatonin profiles in PTSD patients. Other evidence underscores a key function of the melatonergic system in sleep and chronodisruption following trauma exposure and its probable role in a chronically dysregulated circadian network in PTSD (26, 32).

For healthy individuals who sleep at night, daytime endogenous melatonin levels are barely detectable. Once the sun sets, melatonin production begins to rise with a peak near the midpoint of the dark interval (33). This phenomenon is called dim light melatonin onset (DLMO) (34). After sunrise, the nocturnal surge of melatonin ends; called melatonin offset (MelOff) (35, 36). Recent investigations have highlighted the ubiquitous influence of circadian timing and melatonin in almost all physiologic functions (35, 36). An altered sleep-wake cycle has been correlated with physiological imbalances that are linked to the development of various mental disorders and melatonin-based chronobiologic interventions may prove efficacious for treatment of circadian disruption in PTSD (37–39). Moreover, mounting evidence suggests that strong antioxidant activities and diverse immunomodulatory properties of melatonin as potential protective roles in diverse neuropsychiatric disorders including PTSD (38, 40). The aim of the current research was to test the hypothesis that military-related PTSD with clinically endorsed “sleep difficulties” is associated with decreased production of endogenous melatonin and possible chronodisruption, relative to healthy military controls. We investigated whether salivary melatonin levels and sleep patterns are different in Canadian Armed Forces (CAF) military personnel with PTSD when compared to matched healthy CAF members as controls, by assessing daily sleep quality for 7 days and measuring 24-h salivary melatonin secretion on the eighth day of study.

## Materials and Methods

### Study Design

We conducted a retrospective cohort study investigating relationships between sleep disturbances and salivary melatonin levels in CAF military personnel with PTSD and matched healthy CAF members as controls.

### Participants and Clinical Procedures

Volunteers included treatment-seeking CAF members (N = 7) with clinically endorsed “sleep difficulties,” aged 31 to 45 years with mean age and standard deviation of 37.57 ± 6.05. These participants had experienced a self-reported deployment-related trauma with a “current” diagnosis of PTSD by a licensed CFHS (Canadian Forces Health Services) and/or Department of Veteran Affairs of Canada (VAC) psychiatrist. Each diagnosis was based upon a comprehensive psychiatric examination, according to criteria outlined by the Clinician Administered PTSD Scale (CAPS) for the DSM-5, including CAPS score ≥50 (41). Healthy CAF members (N = 14) who had not been diagnosed with any DSM-5 condition (including PTSD) at any point in their lives or reported sleep disorders, served as controls. Controls were stratified according to those whose data was collected under either naturalistic diurnal environmental conditions (NC; N = 7) aged 22 to 52, with mean age and standard deviation of 35.25 ± 9.38 or idealized sleep-wake conditions with strictly controlled nocturnal light-exposure, as previously described (light controlled (LC); N = 7) aged 23 to 43 with mean age and standard deviation of 29.43 ± 8.50 (42). A comparison of all participants is presented in **Table 1**.

**Table 1:** Demographic characteristics of study participants.

Group	Sample size (N)	% Male	Mean age ± SD (range)
PTSD	7	71.4	37.57 ± 6.05 (31–45)
NC	7	85.7	34.14 ± 9.55 (22–54)
LC	7	57.1	29.43 ± 8.50 (23–43)

PTSD, posttraumatic stress disorder; NC, normal controls; LC, light controlled.

Participants were excluded if they: a) reported current suicidal or homicidal ideation; b) met DSM-V criteria for current substance abuse or dependence (except nicotine or caffeine) per self-reported answers on the diagnostic interview; or c) met DSM-V criteria for a psychotic disorder; or d) were diagnosed with incapacitating cognitive impairment, traumatic brain injury; or e) were taking supplementary melatonin; or f) were on specific medications known to alter endogenous melatonin levels (e.g., beta blockers, methamphetamines or nonsteroidal anti-inflammatory drugs); and/or (g) recent shift work or transmeridian travel. Participants were asked to refrain from the use of alcohol for 24 h prior to participation in data collection.

This investigation protocol was approved by the Defence Research and Development Canada (DRDC) Human Research Ethics Committee (HREC; Protocol No. 2017-023) and adhered to the standards and regulations set forth by the Tri-Council Policy in accordance with the Ethical Principles for Medical Research Involving Human Subjects adopted in the Declaration of Helsinki. All participants provided written informed consent after a complete description of the study procedural details were explained to them and discussing any questions or concerns about the protocol. Participants were recruited between 2017 and 2018 by DRDC—Toronto Research Centre (TRC) non-physician research coordinator/staff through referrals by military physicians, mental health professionals, community clinics, and advertisements within Canadian Forces Bases (CFB) and/or through one of several Operational Stress Injury (OSI) clinics of the Department of Veterans Affairs Canada (VAC).

### Experimental Procedures

All participants wore a standard wrist activity monitor (Motionlogger version 14.000, Ambulatory Monitoring Inc., Amherst, NY, USA) for 7 days prior to the data collection to establish their baseline sleep hygiene. The actigraphic data were analyzed by the Cole-Kripke algorithm (43). Parameters assessed during the week prior to the 24-h data collection were daily bedtimes, arise times, time in bed (min), total wake minutes, total sleep minutes, sleep efficiency (%), Sleep LATency (SLAT), wake after sleep onset (WASO), and # of sleep episodes (see **Supplementary Data Table 2**). To assist scoring of the actigraph data all participants maintained a daily sleep log.

On the 8^th^ day, all participants underwent a 24-h salivary melatonin assessment by providing 13 bi-hourly samples commencing at 0900 h and finishing at 0900 h the next day. All saliva samples were collected in very dim light (5 lux) since normal room lighting levels will suppress endogenous melatonin production. Each participant was assigned to a lounge chair for the entire 24-h data collection period. The subjects were allowed to get up from their chairs to access the washrooms or socialize for about 100 min of each 2-h block. However, they remained in ambient lighting levels of less than 5 lux throughout the 24-h data collection. All subjects returned to semi-recumbent posture in their lounge chairs at least 15 min prior to each sample. The saliva was collected in test tubes (Salivette^®^, Starstedt Inc. Montreal, QC). For each sample, the subjects chewed the cotton plug for 45 s, and then stopped chewing with the plug still in their mouth to allow the saliva to absorb for another 45 s. The participants were required to remain awake from 0900 h until 2300 h, after which they were allowed to sleep. If asleep, they were awakened in the 15 min prior to the next sample. Meals were provided in three 1-h windows as follows: 1) lunch 12–13 h, 2) dinner 17–18 h, and 3) breakfast 07–08 h.

The saliva samples were analyzed for melatonin content using the Buhlmann Laboratories (Schönenbuch, Switzerland) enzyme-linked immunosorbent assay (ELISA) kits and used the Kennaway G280 anti-melatonin antibody. Each subject’s samples were analyzed in duplicate on the same ELISA plate. The reported intra- and inter-assay coefficients of variation for the kits were 12.6 and 22.9% respectively, with a limit of detection (sensitivity) of 0.5 pg/ml. Using the same melatonin collection protocol and melatonin ELISA, we compared the current melatonin and sleep findings to melatonin and sleep data from a cohort of seven military personnel while they were deployed to the most northerly manned outpost in the world (CFS Alert) during arctic summer (i.e., 24/7 sunlight) (42). This was the LC group. The PTSD group was assessed at Canadian Forces Base (CFB) Gagetown (Fredericton, New Brunswick). The normal control (NC) group were assessed at DRDC—TRC.

The distribution of military occupation specialties among the PTSD and healthy control groups are illustrated in **Supplementary Data Table 1**.

Of the seven PTSD participants, three were taking the sleeping medication zopiclone, one was taking lorazepam (an anxiolytic known to facilitate sleep), one was taking oxazepam (another anxiolytic known to facilitate sleep), and one was on both zopiclone and trazodone (an antidepressant known to facilitate sleep). Essentially, six of the seven PTSD participants were on medications to facilitate sleep (see **Supplementary Data Table 1**). None of the control participants (NC or LC) were on any medication. The gender split and age demographics of all three participant groups are illustrated in **Table 1**.

### Statistical Analyses

All demographic and clinical data in the tables are expressed as number and percentage of subjects [*N* (%)] or mean and standard deviation [mean (±SD)]. The mean (±SEM) salivary melatonin concentrations were compared using a “group” (PTSD *vs.* NC *vs.* LC) x “time of day” repeated-measures univariate analysis of variance (ANOVA) using the Geisser-Greenhouse correction for repeated measures. The mean (±SEM) of all sleep parameters were compared using a group x day repeated-measures univariate ANOVA to assess the effect of group and days and their interactions on each of the sleep parameters. *Post hoc* comparisons of significant effects in ANOVAs were performed using Newman-Keuls test. A p-value < 0.05 was considered statistically significant. Statistical analyses were performed using STATISTICA Software Version 13, Palo Alto CA, USA.

## Results

### Twenty-Four Hour Melatonin Production

**Figure 1:**
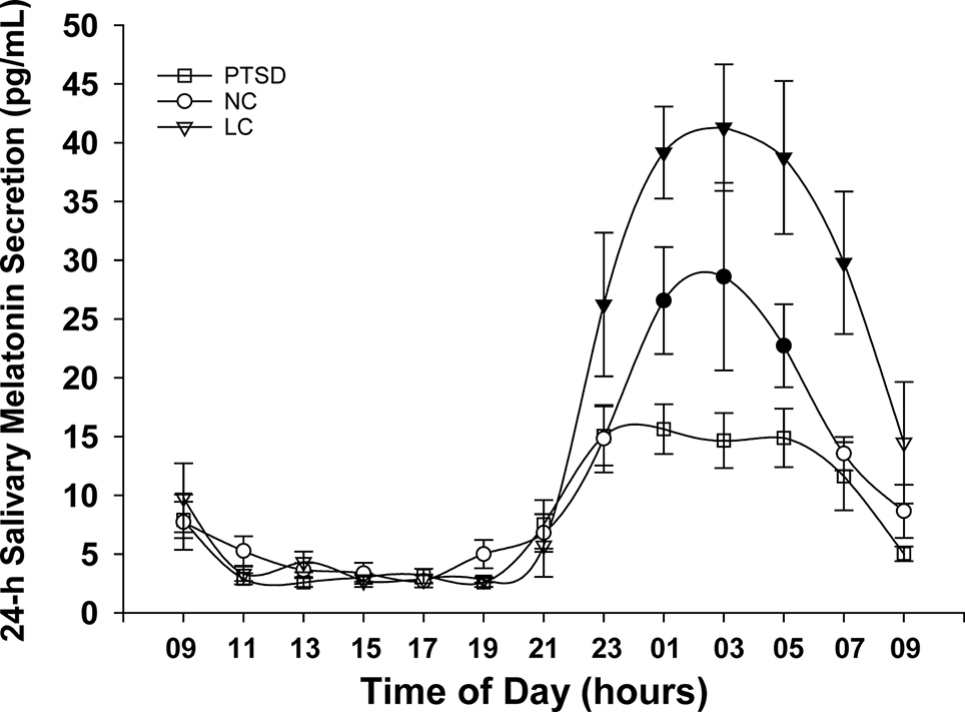
Mean melatonin concentrations (pg/ml) sampled over a 24-h period for each of the three groups: posttraumatic stress disorder (PTSD), normal controls (NC), and light controlled (LC). Significant differences occurred during physiologic night (2300 to 0700 h). Shaded symbols indicate values that are significantly (p < 0.05) different between groups: the time of day data points where NC and LC demonstrate higher melatonin levels than PTSD are indicated with blackened circles (NC) or blackened triangles (LC), respectively. All values are means ± SEM.

**Figure 2:**
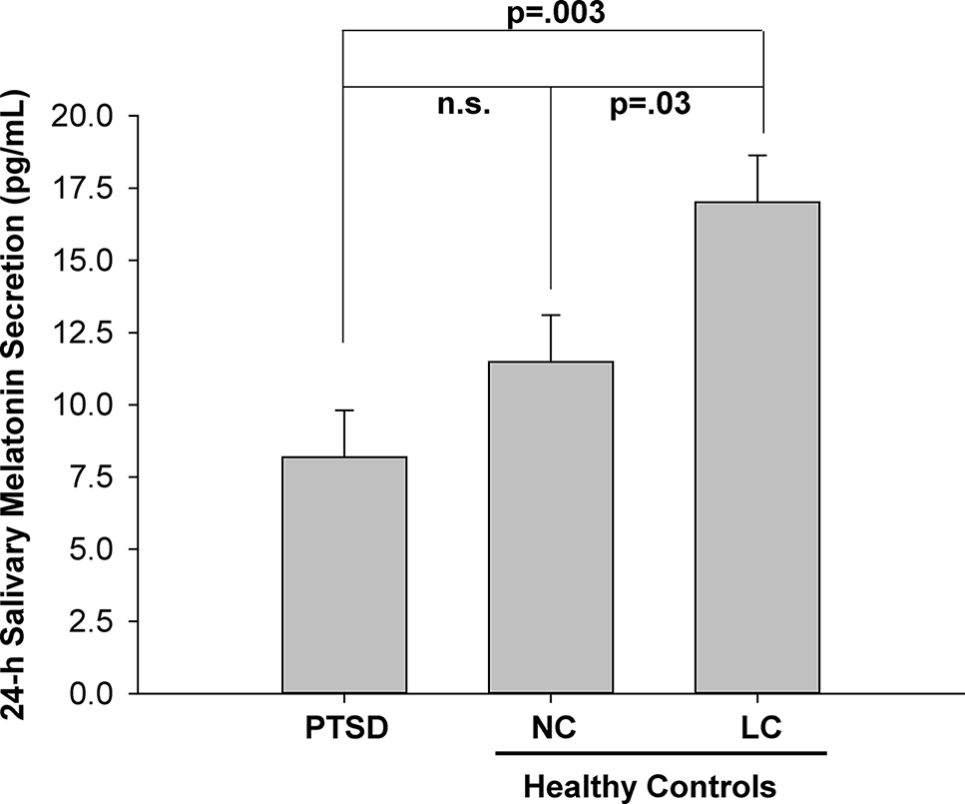
Mean melatonin concentration (pg/ml) for each of three groups: posttraumatic stress disorder (PTSD), normal controls (NC), and light controlled (LC). All values are means ± SEM.

The 24-h salivary melatonin secretion curves for all groups are shown in **Figure 1**. Overall, peak salivary melatonin concentrations (pg/ml ± SEM) were 17.9 ± 1.7, 32.3 ± 7.9, and 58.0 ± 7.0 in the PTSD, NC, and LC groups, respectively. These peak levels occurred between 2300 and 0500 h for each of the PTSD and NC groups and between 0100 and 0500 h for the LC group. The 3 groups x 13 samples ANOVA indicated that the main effect of "group" was significant F(2,18) = 7.52, p = .004 (**Figure 2**), and the group x time of day interaction F(6.93,62.36) = 4.26, p = .001 was significant (**Figure 1**). *Post hoc* testing of the group x time of day interaction confirms that the NC group produced more melatonin than the PTSD group between 0100 and 0500 h, whereas the LC group produced more melatonin than either the NC or PTSD groups between 2300 and 0700 h.

The significant main effect of group (**Figure 2**) indicated that the mean 24-h melatonin production was higher in the LC group than the PTSD and NC groups.

### Sleep Data

#### Posttraumatic Stress Disorder Participants With the Best and Worst Sleep

To sustain optimum health daily sleep should involve approximately 8 h in bed, a regular bedtime, and normal sleep efficiency (80 to 90%). To achieve this sleep quality, sleep latency should be low (generally less than 10–15 min) with minimum WASO, and only a few sleep episodes (44).

The sleep data for all three groups (PTSD, NC, and LC) are illustrated in **Supplementary Data Table 2**.

Participant 2 had the worst sleep of the PTSD group while participant 6 had the best sleep of this group. Participant 2 averaged about 428 min (7 h and 8 min) in bed of which only 318 min (5 h and 18 min) were sleep. His mean WASO was very high (89 min) and his sleep efficiency was low (78.10%). He also averaged a very high number of sleep episodes (36), indicating extremely fractured sleep. This participant was not taking medications that facilitate sleep.

In contrast to participant 2, participant 6 had an average of 456 min (7 h and 36 min) of daily nocturnal sleep. His sleep average efficiency was 94%, and his average sleep latency and WASO were 11 min and 28 min, respectively. His average number of sleep episodes was 14 (upper normal range).

On average, participant 2 took about 19 min longer to fall asleep than participant 6. Participant 2 also spent about 60 min longer awake after initial sleep onset and had about 23 more sleep episodes each night than participant 6. The other PTSD participants (numbers 1, 3, 4, 5, and 7) had sleep quality that was intermediate between the extremes of participants 2 and 6 in all of these parameters.

#### Normal Control Participants With the Best and Worst Sleep

Participant 8 had the worst sleep of the NC group while participant 10 had the best sleep of this group.

Participant 8 reported that his sleep was compromised because he was studying in the evenings. His average daily time in bed was 400 min, resulting in 324 min (5 h and 24 min) of sleep. His sleep efficiency was quite low at 72%. While his average sleep latency 17 min. He averaged about 26 sleep episodes per night, reflecting significantly fractured sleep.

Participant 10 averaged about 533 min (8 h and 53 min) in bed, resulting in an average of 497 min (8 h and 17 min) of sleep. His average sleep efficiency was just over 93%, while his sleep latency and WASO were about 19 and 24 min respectively. His average number of sleep episodes was about 10 per night and within normal limits.

On average, participant 10 achieved about 133 min (2 h and 13 min) more daily time in bed and achieved 173 min (2 h and 53 min) more sleep than participant 8. Participant 10 also had about a 21% higher sleep efficiency than participant 8. On average, participant 8 took about 2 min longer to fall, spent about 68 min longer awake after initial sleep onset, and had about 16 more sleep episodes each night than participant 10. The other NC participants (numbers 9, 11, 12, 14, and 15) had sleep quality intermediate between the extremes of participants 8 and 10 in all of these parameters.

#### Light Controlled Participants With the Worst and Best Sleep

Participant 12A had the worst sleep of the LC group, while participant 1A had the best sleep of this group.

Participant 12A had an average bedtime of 0005 h. He averaged about 406 min (6 h and 46 min) in bed, resulting in an average of 379 min (6 h and 19 min) of daily sleep. His average sleep efficiency was just over 93%, while his sleep latency and WASO were about 10 and 18 min respectively. His average number of sleep episodes was about 10 per night and normal.

Participant 1A had an average bedtime of 2301 h. He averaged about 568 min (9 h and 28 min) in bed, resulting in an average of 513 min (8 h and 33 min) of daily sleep. His average sleep efficiency was about 91%, while his sleep latency and WASO were about 9 and 43 min respectively. His average number of sleep episodes was about 16 per night, which was in the high-normal range.

On average, participant 1A achieved about 162 min (2 h and 42 min) more daily time in bed than participant 12A, achieved 134 min (2 h and 14 min) more sleep than participant 12A and had a similar sleep efficiency (about 91 *vs.* 93%) as participant 12A. Both participant 1A and 12A had very similar sleep latencies (9 *vs.* 10 min). Participant 1A had a higher average WASO than participant 12A (43 min *versus* 88 min) and a higher average number of daily sleep episodes (16 *versus* 10) reflecting lower sleep pressure in participant 1A, since he was well rested. The other LC participants (numbers 6A, 7A, 8A, 10A, and 11A) had a sleep quality that was intermediate between the extremes of participants 1A and 12A in all of these parameters.

Each of the sleep parameters shown in **Supplementary Data Table 2** were analyzed by three groups x 7 days repeated-measures analysis of variance. There were no group differences in any parameter, although there were some differences across days of the week, indicating that all three groups exhibited normal weekend sleep behaviors of later bed times and arise times, and increased sleep minutes.

## Discussion

This is the first study to measure salivary melatonin secretion over a 24 h period in military PTSD cases *versus* matched controls. The melatonin profile curve in the PTSD group showed a blunted nocturnal peak and lower total quantity of melatonin production relative to the healthy control groups (NC and LC). By contrast, there was little difference in the sleep parameters assessed between the three groups.

Since six of seven PTSD participants were either on sleeping medications or anxiolytics that are known to facilitate sleep, the PTSD group sleep characteristics were better than expected. Two participants in the NC group had multiple nights with bedtimes ranging from 0130 to 0330 h, which decreased the average quality of the NC group sleep. The LC group (based in the high arctic) were restricted to quarters after 2000 h for safety due to the presence of polar bears around the CFS Alert Station; hence, they avoided exposure to outdoor 24-7 daylight prior to bedtime. Consequently, their sleep-wake was better than we have measured previously in other participants at CFS Alert during arctic summer (42). The delays in bedtime and arise time that were evident in Saturday and Sunday (for all three groups) simply reflected normal weekend behavior in that there was no obligation to report to work on Saturday or Sunday mornings.

Notwithstanding the similarity in sleep hygiene across the three groups, there were significant differences in nocturnal melatonin production between these groups. Both the NC and PTSD groups produced significantly less overall daily melatonin than the LC group (**Figure 2**). Despite the fact that six of the seven PTSD participants were on medications known to counter insomnia, it is apparent these sleeping medications did not restore the levels of endogenous melatonin for this group. The LC group would normally have had access to the outdoors in the arctic summer evening, which would have suppressed their melatonin, thus delaying their sleep pressure and hence their bedtime, causing difficulty to arise at their normal time the following morning. In this instance, the lack of access to the outdoors precluded the suppression of melatonin that we have seen previously in personnel stationed at CFS Alert during the arctic summer. The amplitude of the nocturnal surge of melatonin in the LC group (**Figure 1**) was quite normal and very similar to the melatonin profiles that we have measured in the lower latitudes across Canada (45). In contrast, the amplitude of the melatonin rhythm in each of NC and PTSD groups is significantly lower. In the case of the NC group, the lower amplitudes were probably related to the sleep behaviors of these participants, several of whom had substantially later bedtimes and likely late evening light exposure as a result.

In the PTSD group, the markedly lower amplitude of the nocturnal surge was likely associated with a pathophysiological response to their PTSD and may signify an inability to maintain an circadian sleep-wake rhythm (30, 46). The duration of the nocturnal surge of endogenous melatonin in the LC group ranges from about 2100 to 0900 h, and is also quite typical and similar to what we have previously measured. In contrast the duration of the nocturnal surge of endogenous melatonin for the NC and PTSD groups was shortened. The shortened duration of the nocturnal melatonin surge of the NC group was likely due to the nocturnal exposure to light (of the subjects who had delayed bedtimes) (47, 48). Similar to the reduced amplitude of melatonin, the shortened duration of the nocturnal melatonin surge of the PTSD group was likely a direct consequence of their PTSD as their sleep behaviors were generally in the normal range due to sleep facilitation medications and thus did not have late evening light exposure (30, 46). Our findings of blunted melatonin production in PTSD, are consistent with those of MacFarlane et al. (30) and may explain the findings of Seyffert and Gettey (38) that supplementary bedtime melatonin was helpful in sleep restoration and a reduction of symptoms in their case study of a veteran with PTSD. Pharmacotherapy, usually selective serotonin reuptake inhibitors (SSRIs), and psychotherapy, such as cognitive-behavioral therapy, are regarded as the first-line treatments for military PTSD (17). However, treatment-refractory PTSD, a more severe form of the illness, has low rates of response or remission, even after an adequate trial of SSRI therapy (16). Thus, finding alternatives to SSRIs for treatment of PTSD is imperative to relieve the suffering associated with this disorder (49)

Melatonin production typically declines with age (50), and more so in neurodegenerative diseases, such as dementia (51). Deficiency in the production of endogenous melatonin that results in specific functional losses are improved by supplementation with exogenous melatonin (52). There is ample evidence of reduced endogenous melatonin in various types of cardiac diseases (53–57), fibromyalgia (51, 58), neuralgia (58), migraine (59, 60), epilepsy (61, 62), Menières disease (63), various cancers (64, 65), attention-deficit hyperactivity disorder (66), recurrent depression (67), and bipolar disorder (68). Agorastos and Lindhorst describe potential pleiotropic benefits of melatonergic treatments to remediate problems with chronodisruption, hypothalamic pituitary adrenal-axis dysfunction, sympathoadrenal and autonomic dysregulation, neuroimmunomodulation, oxidative stress, brain injury, cognitive function, memory and neurocircuitry, mood and anxiety disorders (32). The work reported here indicates that PTSD should be added to this evolving list of disorders/diseases that result in blunted production of endogenous melatonin, and work investigating the efficiency of supplementation with exogenous melatonin in PTSD sufferers should be undertaken soonest. There is also recent evidence that long-chain polyunsaturated fatty acids can be beneficial in attenuating PTSD symptomology (69). These and other antioxidant strategies for treating PTSD should also be investigated.

The results of this vanguard study must be interpreted within the context of its limitations. We recognize that this study suffers from a small sample size, and that larger military cohort studies are required to expand and more conclusively confirm these initial findings. However, even with a modest sample size of seven PTSD cases, this preliminary evidence is quite compelling with respect to the dramatically reduced levels of melatonin in the PTSD cohort. Similarly, our military control sample was not ideal, since a military career and operational requirements impacted the sleep/wake cycle of these individuals. Future studies involving military populations must recognize that the less than ideal sleep habits often observed in serving military members may influence or even confound their results for sleep parameters. Thus, investigators may consider, as we did, strictly controlling nocturnal light exposure, imposing daily bedtime deadlines and encouraging sufficient daily time in bed to obtain optimal results for melatonin production and calculation of DLMO.

## Conclusion

Our findings reveal severely blunted nocturnal melatonin secretion in those individuals with military-related PTSD. Based upon these findings, further investigations through preclinical research and clinical interventional studies are warranted. This may include methodologically robust prospective randomized controlled studies, assessing the efficacy of melatonin treatment strategies in the prevention and/or treatment of PTSD. Given the potential pleiotropic benefits of melatonergic therapies to remediate problems with chronodisruption and neuroimmunomodulation, melatonin treatment could provide a potentially promising therapeutic approach with beneficial effects in the management of PTSD and trauma-related syndromes and comorbidities. In particular, therapeutic doses of melatonin may be beneficial as a complementary or alternative approach in those subgroups of combat veterans with severe sleep abnormalities and who are refractory to commonly employed psychotherapeutic and/or pharmacological options.

## Data Availability Statement

The datasets generated for this study are available on request to the corresponding author.

## Ethics Statement

The studies involving human participants were reviewed and approved by Defence Research and Development Canada (DRDC) - Human Research Ethics Committee (HREC). The patients/participants provided their written informed consent to participate in this study.

## Author Contributions

All authors were involved in the drafting and revising of the manuscript, as well as the interpretation of the data. MP, RL, and SR all contributed substantially to the conception and design of this work. MP, RL, and SR contributed substantially to the acquisition and analysis of the data.

## Conflict of Interest

The authors declare that the research was conducted in the absence of any commercial or financial relationships that could be construed as a potential conflict of interest.

## Supplementary Material

Supplementary Table 1 PTSD exposure/chronicity, medications, military rank of study participants.

Supplementary Table 2
Daily sleep statistics (all parameters are means over 7 days of sleep) for participants with the best and worst sleep for each of the Posttraumatic stress disorder (PTSD), normal controls (NC), and light controlled (LC) groups.
