# Supplementary material for: Blunted Nocturnal Salivary Melatonin Secretion Profiles in Military-Related Posttraumatic Stress Disorder
Source: Front Psychiatry. 2019 Dec 6;10:882. doi: 10.3389/fpsyt.2019.00882 (PMC6910089; doi:10.3389/fpsyt.2019.00882)
Supplement: Supplementary Table 1 — PTSD exposure/chronicity, medications, military rank of study participants. [file DataSheet_1.docx]

**Supplementary Data**

**Supplementary Table 1.** PTSD exposure/chronicity, medications, military rank of study participants

| ***Subject #*** | ***Exposure to trauma*** | ***Symptom onset*** | ***PTSD diagnosis*** | ***Data collection date*** | ***On sleeping meds or anxiolytics?*** | ***MOC*** | ***Rank*** | ***Gender*** |
| --- | --- | --- | --- | --- | --- | --- | --- | --- |
| 1 | 2006-2007 | Immediate | 2017 | Late Nov 2017 | Zopiclone, Desvenlafaxine | Infantry | MCpl | Male |
| 2 | 2006 and 2012 | 2013 | 2017 | Late Nov 2017 | No | Sniper | MCpl | Male |
| 3 | 2006 and 2008 | Immediate | 2014 | Late Nov 2017 | Lorazepam | Artillery | WO | Male |
| 4 | 2009-2010 | Immediate | 2010 | Late Nov 2017 | Zopiclone | Combat engineer | SGT | Male |
| 5 | 2016 | Immediate | 2017 | Late Nov 2017 | Zopciolone | Radio Operator | WO | Female |
| 6 | 2002 | 2016 | 2017 | Late Nov 2017 | Oxazepam | Lineman | WO | Male |
| 7 | 2010 | 2014 | 2017 | Late Nov 2017 | Zopiclone, Trazodone | Supply Tech | MCpl | Female |

**Supplementary Table 2**

***Supplementary Table 2.***  *Daily sleep statistics (all parameters are means* *over 7 days of sleep) for participants with the best and worst sleep for each of the Posttraumatic stress disorder (PTSD), normal controls (NC), and light controlled (LC) groups*

|  | | **bed-time** | **arise time** | **time in bed (min)** | **wake min** | **sleep min** | **sleep efficiency (%)** | **sleep latency (min)** | **WASO (min)** | **# of sleep episodes** |
| --- | --- | --- | --- | --- | --- | --- | --- | --- | --- | --- |
| **PTSD** | Subject 2 worst sleep | 00:02:43 | 6:56:07 | 427.57 | 109.86 | 317.71 | 78.10 | 30.43 | 89.14 | 37.25 |
|  | Subject 6 best sleep | 22:02:19 | 6:17:32 | 494.14 | 38.71 | 456.43 | 94.08 | 11.14 | 28.29 | 14.14 |
| **NC** | Subject 8 worst sleep | 02:06:00 | 8:54:00 | 399.75 | 110.25 | 323.75 | 72.01 | 17.00 | 91.63 | 25.75 |
|  | Subject 10 best sleep | 22:46:22 | 7:40:28 | 533.38 | 37.88 | 496.50 | 93.39 | 18.88 | 23.50 | 9.88 |
| **LC** | Subject 12A worst sleep | 00:05:00 | 6:51:00 | 406.29 | 28.00 | 379.14 | 93.19 | 10.14 | 18.43 | 9.71 |
|  | Subject 1A best sleep | 23:01:00 | 8:28 | 568.00 | 54.71 | 513.43 | 90.66 | 9.14 | 43.43 | 15.71 |
